# Supplementary figures and images for: Sirt6-Mediated Cell Death Associated with Sirt1 Suppression in Gastric Cancer
Source: Cancers (Basel). 2024 Jan 16;16(2):387. doi: 10.3390/cancers16020387 (PMC10814469; doi:10.3390/cancers16020387)

Fig. 2

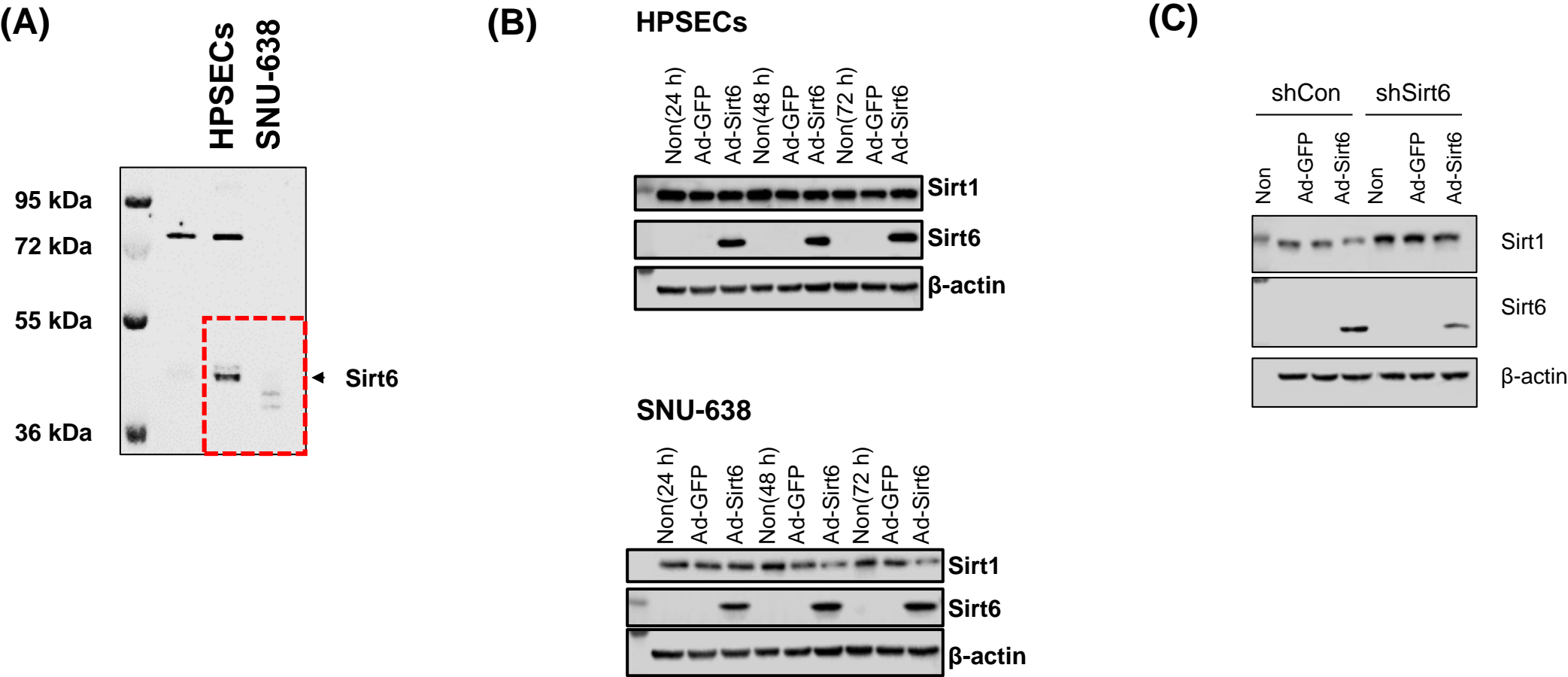

**Fig. 4**

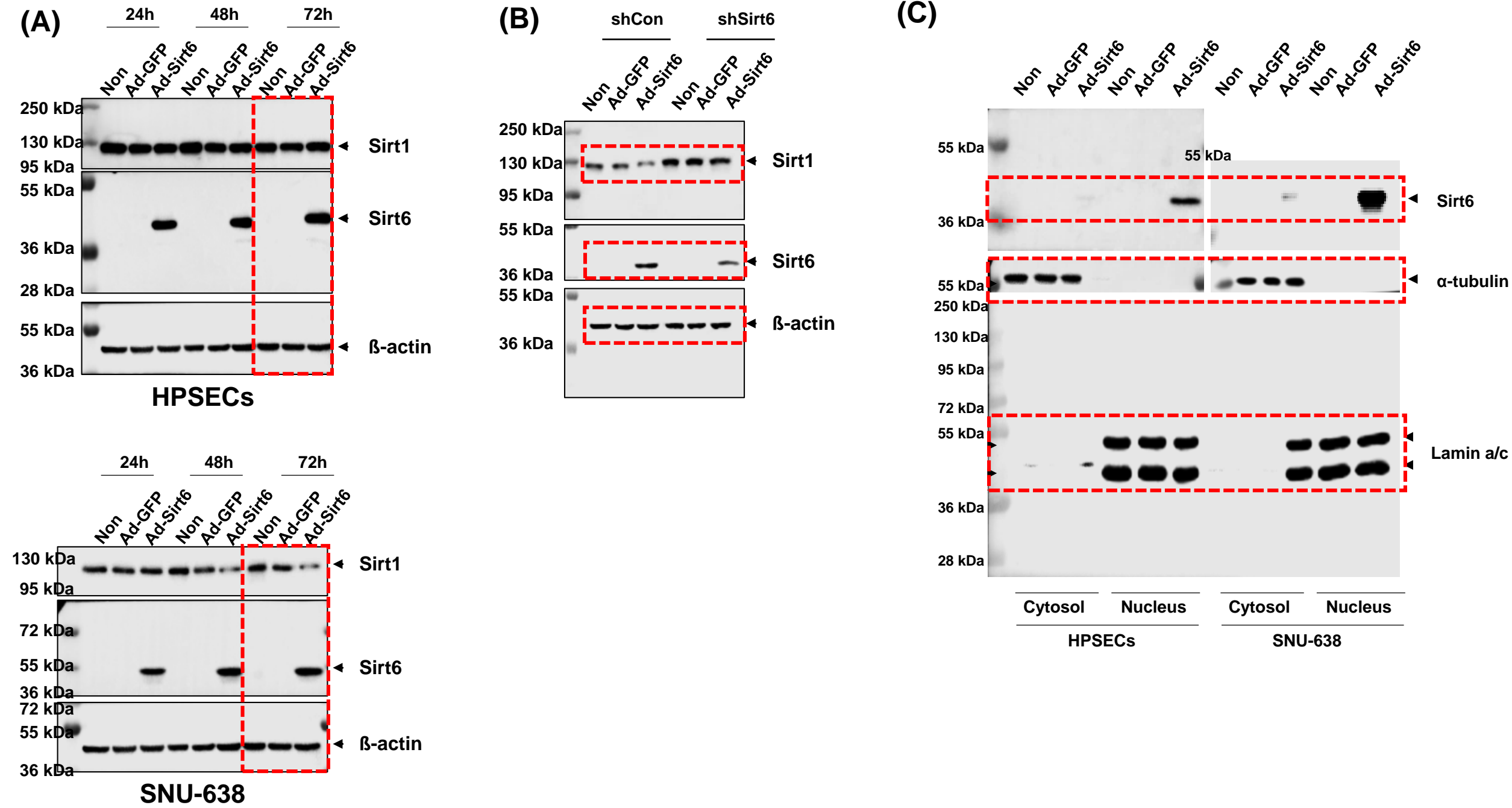

Fig. 5

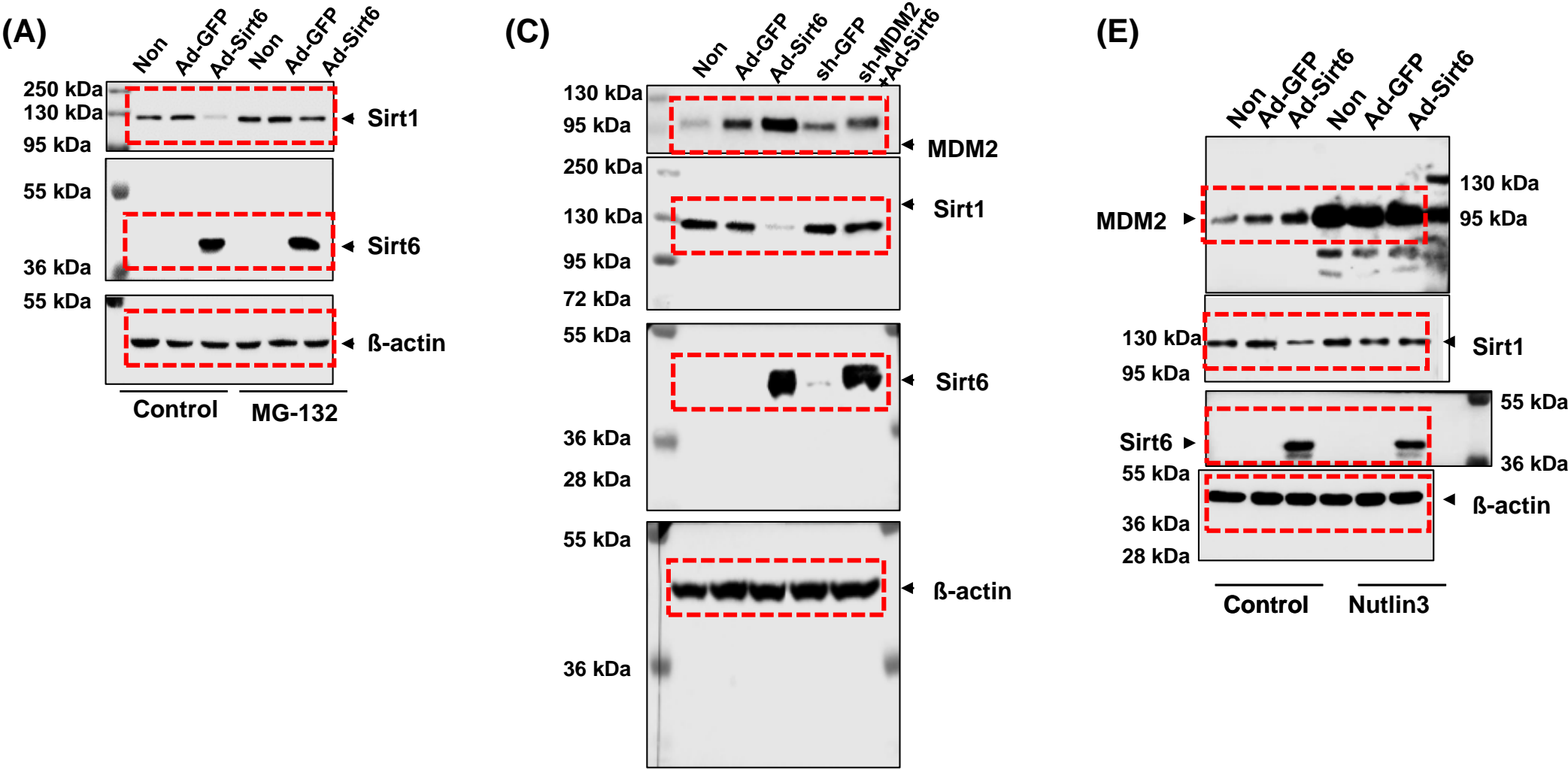

Fig. 5

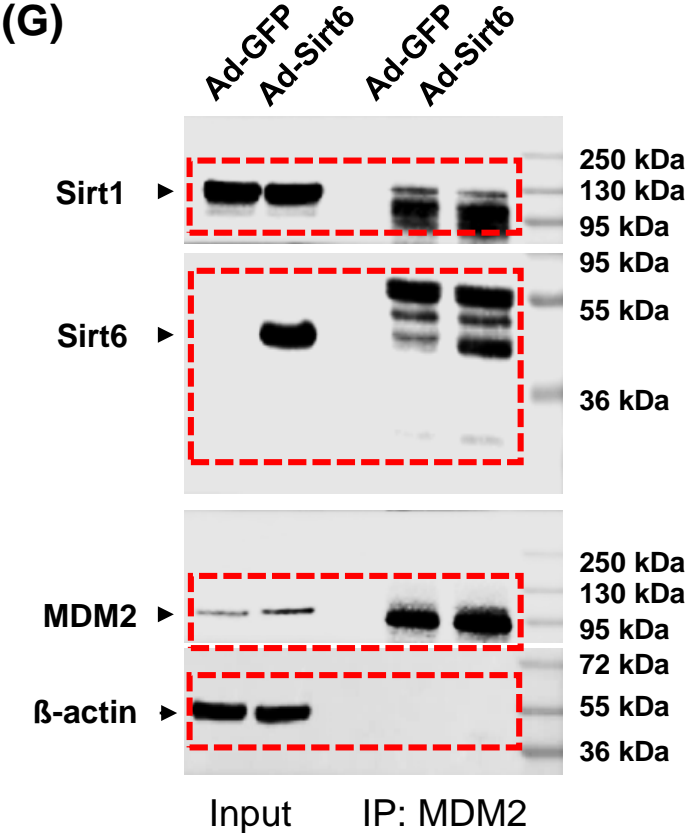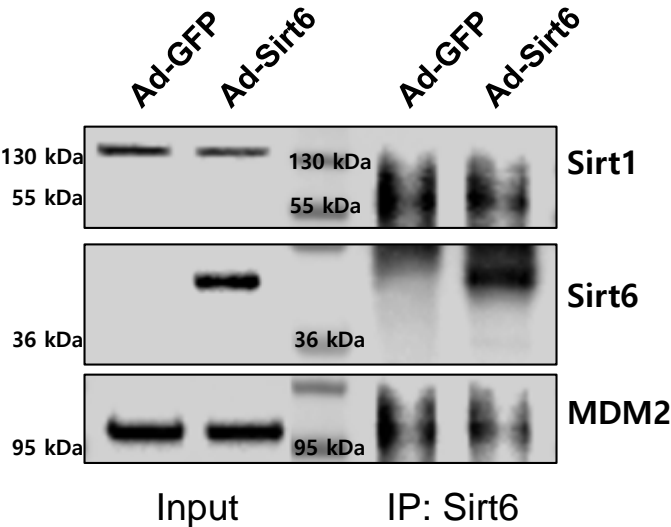

Fig. 4

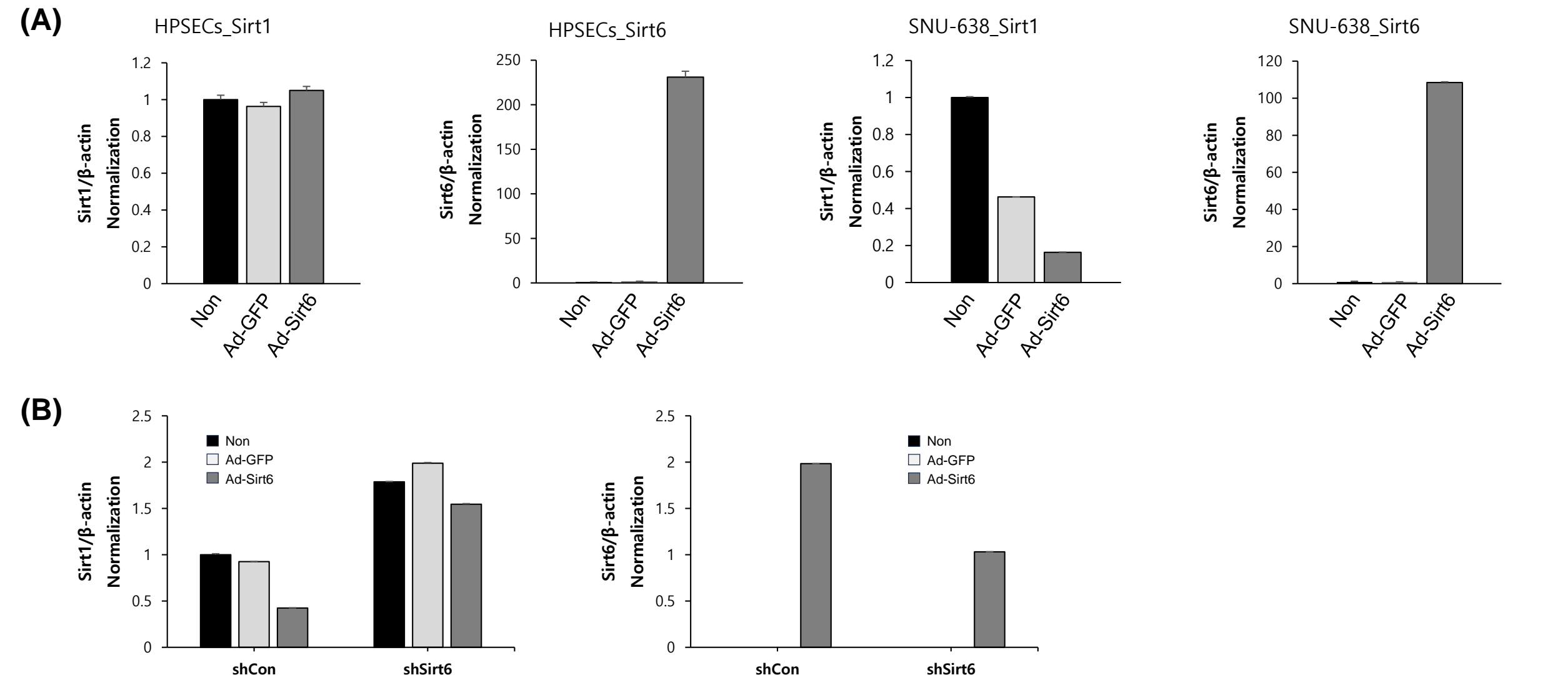

**Fig. 5**

**(A)**

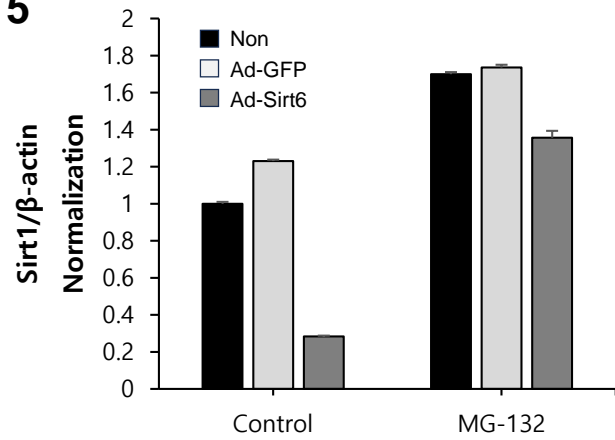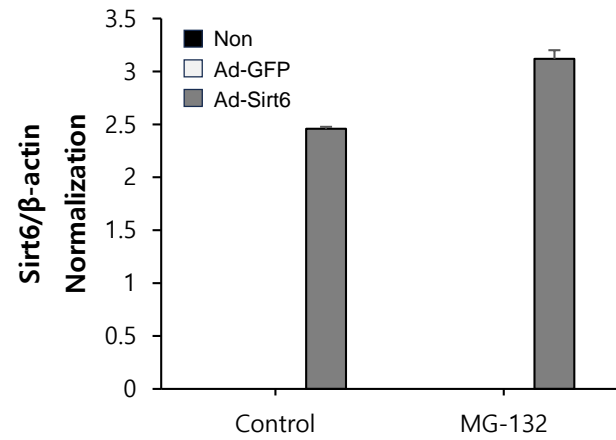

**(C)**

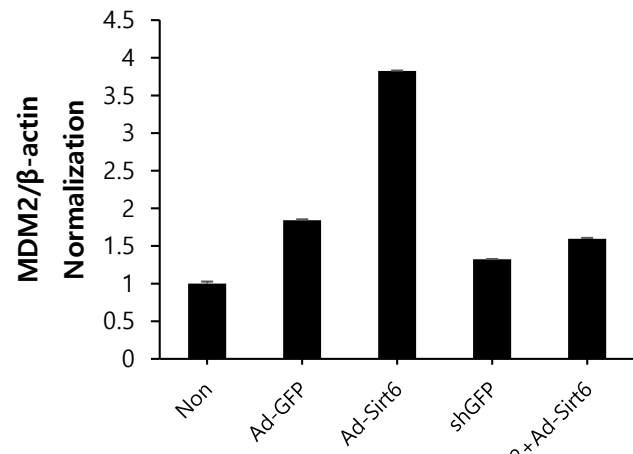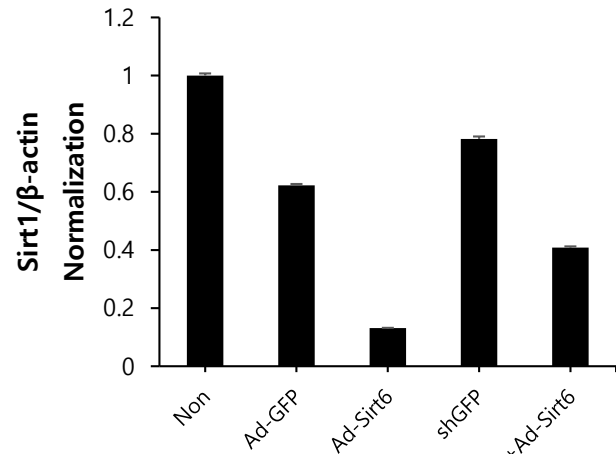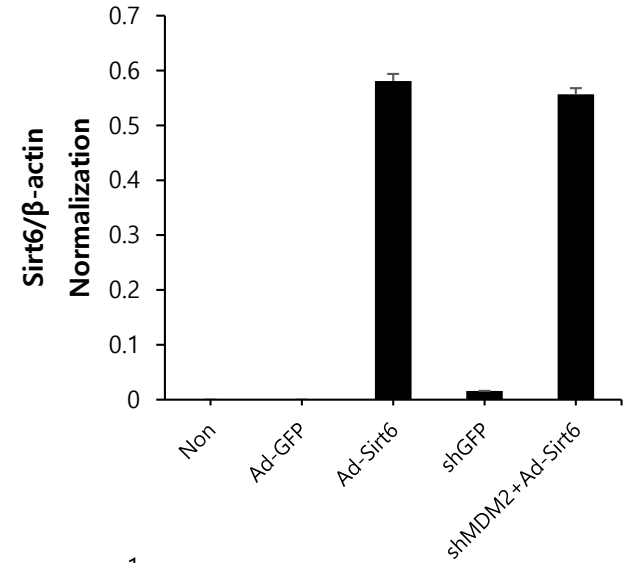

**(E)**

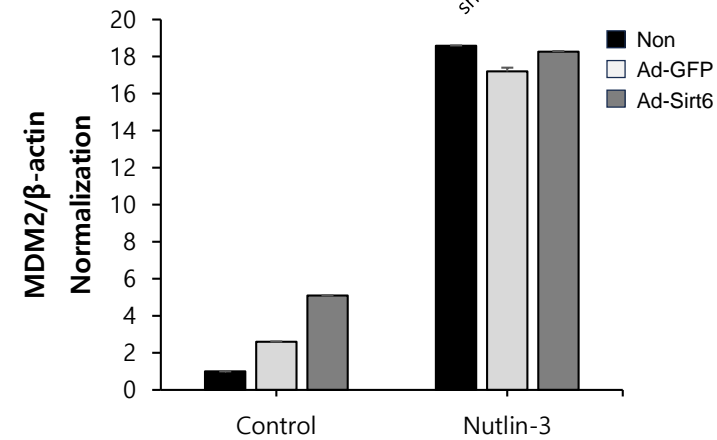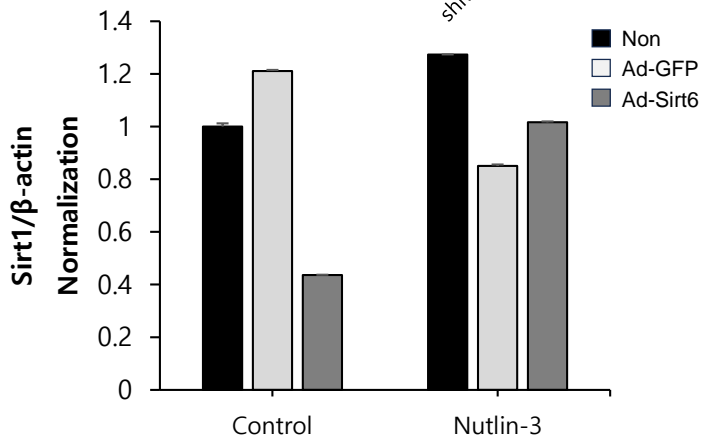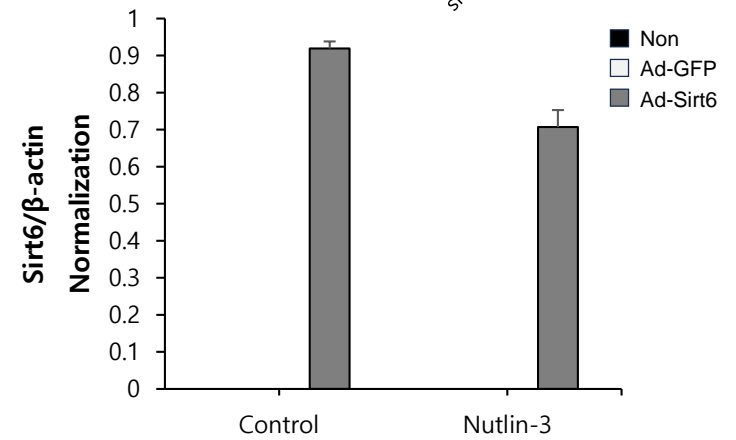

Supplement: Supplementary file 1 [file cancers-16-00387-s001.zip › cancers-2804767-supplementary.pdf]
